# Changes in Population Dynamics in Mutualistic *versus* Pathogenic Viruses

**DOI:** 10.3390/v3010012

**Published:** 2011-01-17

**Authors:** Marilyn J. Roossinck

**Affiliations:** Plant Biology Division, The Samuel Roberts Noble Foundation, Inc., P.O. Box 2180, Ardmore, OK 73402, USA; E-Mail: mroossinck@noble.org; Tel.: +1 580 224 6600; Fax: +1 580 224 6692

**Keywords:** beneficial viruses, polymerase fidelity, quasispecies, symbiosis, symbiogenesis

## Abstract

Although generally regarded as pathogens, viruses can also be mutualists. A number of examples of extreme mutualism (*i.e.*, symbiogenesis) have been well studied. Other examples of mutualism are less common, but this is likely because viruses have rarely been thought of as having any beneficial effects on their hosts. The effect of mutualism on the population dynamics of viruses is a topic that has not been addressed experimentally. However, the potential for understanding mutualism and how a virus might become a mutualist may be elucidated by understanding these dynamics.

## 1. Introduction

Viruses have been studied predominantly as pathogens, beginning with the first virus ever described, *Tobacco mosaic virus* [1] that was causing spots on tobacco plants. However, a number of viruses in plants, animals, fungi and bacteria have been described that are not pathogens; many are commensals and some are mutualists. Traditionally, mutualistic symbioses are thought of as long-term stable relationships, but viruses can clearly switch lifestyles depending on conditions. What effect does mutualism have on the population dynamics of a virus? Do the population dynamics of conditional mutualists change depending on their lifestyle? This is the subject of this brief perspective. It is not intended to be a comprehensive review, but rather to provoke some thought about how the dynamics of virus populations might change when viruses have different lifestyles. For a more comprehensive review on mutualistic viruses the readers are directed to [2].

## 2. Symbiogenic Viruses

Symbiogenesis is the process whereby symbiotic entities fuse and create a new species. This process was first recognized in the discovery of the relationship between mitochondria and bacteria, and is now recognized as an important evolutionary force that may be responsible for major leaps that cannot be explained by Darwinian natural selection [3], and that has probably played a major role in the evolution of new viruses [4,5]. The most extreme cases of virus mutualism are really symbiogenic relationships.

### 2.1. Polydnaviruses

The most well studied mutualistic viruses are the polydnaviruses (poly DNA viruses) [6]. The polydnaviruses are obligate symbionts of their braconid (bracoviruses) or ichneumonid (ichnoviruses) parasitoid wasp hosts. The viruses are required for the successful development of the wasp eggs in the insect hosts that they parasitize [7]. They have been associated with the wasps for so long that some question if they should still be considered viruses [8,9]. Most of the viral genes reside in the nuclear genome of the wasp, while the virions package wasp genes for delivery into the caterpillar where the wasp deposits its eggs. These wasp genes suppress the immune response of the caterpillar and prevent encapsulation of the egg by the caterpillar [7]. Recently, the bracovirus relationship to the insect nudiviruses was demonstrated [10], and the ichnovirus relationship to an as yet unclassified insect virus [11] was also suggested [12]. The most likely scenario for the evolution of the mutualistic relationship is that the viruses were first acquired from insects that the wasps parasitized. The wasps were likely a vector for the insect viruses. This is supported by the characterization of an ascovirus in ichneumonid wasps that has become a mutualist of the wasp that parasitizes its insect host [13]. Unfortunately, no studies have compared the population dynamics of this virus in the wasp where it is a mutualist *versus* the insect where it is a pathogen.

### 2.2. Endogenous Retroviruses

The endogenous retroviruses that make up large portions of many eukaryotic genomes [14] are another example of symbiogenic viruses. Endogenous retroviruses constitute a very large topic (see [2] and references therein); however, the best example of endogenization leading to speciation is in the evolution of placental mammals [15–17]. There are also speculations that endogenous (and exogenous) retroviruses played a role in the evolution of adaptive immunity [18]. There are many other endogenous retroviruses that play a beneficial role in their hosts including protection from pathogens [2]. In addition, in plants the pararetroviruses can protect their hosts from pathogen infections [19].

For the endogenous viruses, there is very limited population variation for the virus outside of the dynamics of the host, since their genomes are replicated as part of the host genome. Hence, in this most extreme case of mutualism the viral populations probably vary only as much as their hosts vary.

## 3. Epigenomic Mutualistic Viruses

For non-endogenous mutualistic viruses population studies are very limited. Some viruses, such as acute RNA viruses of plants that confer drought and cold tolerance, are single-stranded (ss) RNA viruses [20], and are known to have rather large populations within their hosts [21]. However, these viruses have a primary lifestyle that is not mutualistic, but rather pathogenic, so their population dynamics are probably driven by the pathogenic lifestyle. The mutualistic fungal virus, Curvularia thermal tolerance virus (CThTV), is an obligate partner in a three-way symbiosis involving the virus, a fungus and a plant [22]. The holobiont, first discovered in Yellowstone National Park [23], is tolerant of extremely high soil temperatures. This virus has no detectable population variation when grown in its fungal host in culture [24]; the population dynamics of the virus in the intact holobiont have not been studied. However, in addition to being a mutualist, CThTV is a persistent virus. The population dynamics could be driven by either of these forces, or a third, its double-stranded (ds) RNA genome (see below).

Although there are a number of other examples of epigenomic mutualistic viruses [2], there have been few reported studies on the population dynamics of these viruses.

## 4. Within-host Dynamics

### 4.1. Quasispecies

There have been volumes written in recent years about virus populations within individual hosts (see for example [25,26]); for ssRNA viruses these populations are often called quasispecies, and the level of variation can be extreme. It seems likely that ssDNA viruses develop similar diverse populations [27].

### 4.2. Replicase Fidelity

The major source of variation comes from errors made during replication, although chemical mutagens and recombination may also contribute. A number of studies have estimated the fidelity of ssRNA virus polymerases [28], and more recently of a viroid [29]. For ssRNA viruses the polymerases are estimated to make an error about once in 10^4^ nucleotides. For viroids this is much higher, one in between 10^3^ and 10^2^ nucleotides. The fidelity of dsRNA virus polymerases has only been measured for the bacteriophage ϕ6 and it is in the range of one in 10^6^ [30]. Studies with this virus suggest that replication occurs in a stamping machine method, such that all the progeny in a burst are derived directly from the infecting virus genome [30]. It seems unlikely that ssRNA viruses follow this type of replication [31], which would further serve to limit the amount of variation in a population. For viruses that replicate in a geometric fashion, polymerase fidelity is very difficult to measure and for the most part has been approximated. There is only one study of polymerase fidelity directly measured in an intact host, and this only measured indels [32]. Substitutions have not been measured directly.

## 5. Emerging Viruses and Persistence

### 5.1. Emerging and Acute Viruses

Many emerging viruses have ancestors that are not pathogens, but rather persistent viruses of other hosts. A classic example is Human immunodeficiency virus (HIV) that apparently emerged numerous times from the closely related Simian immunodeficiency virus, endemic in chimpanzees [33] and only rarely pathogenic [34], and more recently Severe acute respiratory syndrome virus (SARS) that apparently emerged from wild civet cats [35,36]. Even Influenza virus, which has been a human pathogen for a long time, has new strains that emerge periodically from wild waterfowl populations, usually via a secondary domesticated host like swine [37–39]. In the wild waterfowl the virus does not cause disease. In wild animals these viruses would be called persistent viruses.

There are no studies that directly compare within-host populations of viruses that are both pathogens and mutualists under different circumstances. There is a general conception that quasispecies are large in recently emerged viruses like HIV that are still adapting to their hosts, or in highly virulent pathogens. In poliovirus, greater quasispecies variation correlated with increased virulence [40]. However, in West Nile virus the opposite was found [41]. Hence there is no general model for a correlation between population variation and virulence.

### 5.2. Persistent Viruses

Virtually all life forms that have been examined carry persistent viruses [42,43]. Definitions vary somewhat for persistence in animal hosts, where this term generally refers to a long-term or lifetime infection, in plants and fungi, where persistent viruses are vertically transmitted and remain with the host indefinitely (*i.e.*, though many generations), and in bacteria where persistent viruses are usually lysogenic (*i.e.*, incorporated into the host genome until they excise in a lytic phase). However, in all cases persistent viruses rarely cause detectable disease, and may provide significant benefit to their hosts, either by providing additional functional proteins, or by preventing infection by related acute viruses [43,44].

The effect of persistence on virus population dynamics is almost unknown. In one study of mouse hepatitis virus no population variation was detected in persistent infections, contrasting with acute infections that have a quasispecies nature [45]. This is an intriguing finding that merits some thought. If virulence is associated with high levels of variation, then a commensal or mutualistic virus might be more likely to maintain its lifestyle if its variation level is kept low. This implies something beyond random error-prone virus replication controlling the degree of variation in a quasispecies. Purifying selection may be stronger in these viruses, such that mutants are not tolerated.

The plant persistent viruses are very poorly studied, but it seems quite likely that they provide other essential functions in an epigenetic manner. At least one persistent plant virus, *White clover cryptic virus*, encodes a gene for its legume plant host that can affect nodulation [46], and hence is a mutualist. Additional similar relationships may be discovered in EST libraries from plants, where some ESTs do not have any matches in their cognizant plant genome. However, only a subset of known persistent viruses in plants use poly-adenylation as a strategy to stabilize their RNAs; since most EST libraries are based on poly-A enriched RNA, they may not turn up.

Plant persistent viruses have two features of interest when considering their population dynamics: they all have relatives that are viruses of fungi; and they all have dsRNA genomes. Only one fungal virus population, CThTV, has been studied. However, the amount of population variation in viruses with dsRNA genomes is an interesting question. In fungi almost all the known viruses have dsRNA genomes, and are persistent [47]. A few are known that cause some disease in their fungal hosts [47], and at least one, CThTV, is clearly a mutualist, but the vast majority seem to play no defined role in the lives of their hosts.

## 6. Common Themes

For plant and fungal viruses, persistence and/or mutualism have been found in viruses with dsRNA genomes. The exception is the conditional mutualism of ssRNA acute plant viruses in plants found under extreme conditions of drought or cold [20]. For animals, most of the mutualistic viruses are dsDNA viruses that tend to have much lower population variation, although persistent viruses in animals can have any genome type. The ssRNA viruses described as mutualists are generally conditional mutualists, so their populations may vary with their lifestyles. One well-defined RNA virus mutualism outside of plants and fungi also involves a dsRNA virus, a reovirus that is a mutualist of a parasitic wasp [48]. In many other viral mutualisms, the viruses are either symbiogenic viruses or dsDNA viruses [2], where population variation is naturally more limited. If the limited amount that is known about dsRNA populations (high fidelity replication and very low levels of population diversity) is a general theme for these viruses, then it may be that this is more than coincidence: limited population variation may favor mutualistic relationships. Persistent and mutualistic viruses may be the only types of viruses that could truly be said to have co-evolved with their hosts (*i.e.*, engaged in an arms-race). This could impose significantly more stringent selection pressures that would favor less variation. At this early stage in analyzing the population dynamics of mutualistic viruses, there is a correlation between mutualism and low levels of in-host diversity. However, it is almost certain that we have only begun to scratch the surface of the mutualistic relationships between viruses and their hosts, and the relationships that we do know about have not been carefully examined for population dynamics. Clearly a lot more work is required in this exciting area of research.

## References

[1] Beijerinck MW, Johnson J (1898). Concerning a contagium vivum fluidum as cause of the spot disease of tobacco leaves. Phylopathological Classics, No 7.

[2] Roossinck MJ (2011). The good viruses: Viral mutualistic symbioses. Nature Rev Micro.

[3] Carrapiço F, Rodrigues T (2005). Symbiogenesis and the early evolution of life. Proc SPIE.

[4] Roossinck MJ (2005). Symbiosis *versus* competition in the evolution of plant RNA viruses. Nature Rev Micro.

[5] Roossinck MJ, Roossinck MJ (2008). Symbiosis, mutualism and symbiogenesis. Plant Virus Evolution.

[6] Turnbull M, Webb B (2002). Perspectives on polydnavirus origins and evolution. Adv Virus Res.

[7] Webb BA, Miller LK, Ball LA (1998). Polydnavirus biology, genome structure, and evolution. The Insect Viruses.

[8] Whitfield JB, Asgari S (2003). Virus or not? Phylogenetics of polydnaviruses and their wasp carriers. J Insect Phys.

[9] Stoltz DB, Whitfield JB (2009). Making nice with viruses. Science.

[10] Bézier A, Annaheim M, Herbinière J, Wetterwald C, Gyapay G, Bernard-Samain S, Wincker P, Roditi I, Heller M, Belghazi M (2009). Polydnaviruses of braconid wasps derive from an ancestral nudivirus. Science.

[11] Volkoff A-N, Jouan V, Urbach S, Samain S, Bergoin M, Wincker P, Demettre E, Cousserans F, Provost B, Coulibaly F (2010). Analysis of virion structural components reveals vestiges of the ancestral ichnovirus genome. PLoS Pathog.

[12] Bigot Y, Samain S, Augé-Gouillou C, Federici BA (2008). Molecular evidence for the evolution of ichnoviruses from ascoviruses by symbiogenesis. BMC Evol Biol.

[13] Stasiak K, Renault S, Federici BA, Bigot Y (2005). Characteristics of pathogenic and mutualistic relationships of ascoviruses in field populations of parasitoid wasps. J Insect Physiol.

[14] Lander ES, Linton LM, Birren B, Nusbaum C, Zody MC, Baldwin J, Devon K, Dewar K, Doyle M, FitzHugh W (2001). Initial sequencing and analysis of the human genome. Nature.

[15] Dunlap KA, Palmarini M, Varela M, Burghardt RC, Hayashi K, Farmer JL, Spencer TE (2006). Endogenous retroviruses regulate periimplantation placental growth and differentiation. Proc Natl Acad Sci U S A.

[16] Harris JR (1991). The evolution of placental mammals. FEBS Lett.

[17] Harris JR (1998). Placental endogenous retrovirus (ERV): Structural, functional, and evolutionary significance. Bioessays.

[18] Villarreal LP (2009). The source of self: Genetic parasites and the origin of adaptive immunity. Ann NY Acad Sci.

[19] Staginnus C, Gregor W, Mette MF, Teo CH, Borroto-Fernández EG, Machado MLdC, Matzke M, Schwarzacher T (2007). Endogenous pararetroviral sequences in tomato (*Solanum lycopersicum*) and related species. BMC Plant Biol.

[20] Xu P, Chen F, Mannas JP, Feldman T, Sumner LW, Roossinck MJ (2008). Virus infection improves drought tolerance. New Phytol.

[21] Pita JS, Roossinck MJ, Roossinck MJ (2008). Virus populations, mutation rates and frequencies. Plant Virus Evolution.

[22] Márquez LM, Redman RS, Rodriguez RJ, Roossinck MJ (2007). A virus in a fungus in a plant— three way symbiosis required for thermal tolerance. Science.

[23] Redman RS, Sheehan KB, Stout RG, Rodriguez RJ, Henson JM (2002). Thermotholerance generated by plant/fungal symbiosis. Science.

[24] Roossinck MJ (2011). Population variation in a persistent fungal virus To be submitted for publication.

[25] Domingo E Quasispecies: Concepts and Implications for Virology Springer-Verlag Berlin, Germany 2006 299407

[26] Domingo E (2009). Quasispecies: From molecular Darwinism to viral diseases. Contrib Sci.

[27] Ge L, Zhang J, Zhou X, Li H (2007). Genetic structure and population variability of tomato yellow leaf curl China virus. J Virol.

[28] Duffy S, Shackelton LA, Holmes EC (2008). Rates of evolutionary change in viruses: patterns and determinants. Nat Rev Genet.

[29] Gago S, Elena S, Flores R, Sanjuán R (2009). Extremely high mutation rate of a hammerhead viroid. Science.

[30] Chao L, Rang CU, Wong LE (2002). Distribution of spontaneous mutants and inferences about the replication mode of the RNA bacteriophage ø6. J Virol.

[31] Roossinck MJ, Domingo E, Parrish CR, Holland JJ (2008). Mutant clouds and bottleneck events in plant virus evolution. Origin and Evolution of Viruses.

[32] Pita JS, de Miranda JR, Schneider WL, Roossinck MJ (2007). Environment determines fidelity for an RNA virus replicase. J Virol.

[33] Gao G, Bailes E, Robertson DL, Chen Y, Rodenburg CM, Michael SF, Cummins LB, Arthur LO, Peeters M, Shaw GM, Sharp PM, Hahn BH (1999). Origin of HIV-1 in the chimpanzee. Pan troglodytes troglodytes Nature.

[34] Keele BF, Jones JH, Terio KA, Estes JD, Rudicell RS, Wilson ML, Li Y, Learn GH, Beasley TM, Schumacher-Stankey J (2009). Increased mortality and AIDS-like immunopathology in wild chimpanzees infected with SIVcpz. Nature.

[35] Holmes EC, Rambaut A (2004). Viral evolution and the emergence of SARS coronavirus. Phil Trans R Soc Lond B.

[36] Lipsitch M, Cohen T, Cooper B, Robins JM, Ma S, James L, Gopalakrishna G, Chew SK, Tan CC, Samore MH, Fisman D, Murray M (2003). Transmission dynamics and control of severe acute respiratory syndrome. Science.

[37] Gorman OT, Bean WJ, Webster RG, Holland JJ (1992). Evolutionary processes in influenza viruses: Divergence, rapid evolution, and stasis. Genetic Diversity of RNA Viruses.

[38] Webster RG, Bean WJ, Gorman OT, Gibbs AJ, Calishe CH, Garcia-Arenal F (1995). Evolution of influenza viruses. Molecular Basis of Viral Evolution.

[39] Cohen J, Enserink M (2009). As swine flu circles globe, scientists grapple with basic questions. Science.

[40] Vignuzzi M, Stone JK, Arnold JJ, Cameron CE, Andino R (2006). Quasispecies diversity determines pathogenesis through cooperative interactions in a viral population. Nature.

[41] Jerzak GVS, Bernard K, Kramer LD, Shi P-Y, Ebel GD (2007). The West Nile virus mutant spectrum is host-dependant and a determinant of mortality in mice. Virology.

[42] Villarreal LP (2007). VIrus-host symbiosis mediated by persistence. Symbiosis.

[43] Villarreal LP (2009). Persistence pays: How viruses promote host group survival. Curr Op Microbiol.

[44] Roossinck MJ (2010). Lifestyles of plant viruses. Phil Trans R Soc B.

[45] Stühler A, Flory E, Wege H, Lassmann H, Wege H (1997). No evidence for quasispecies populations during persistence of the coronavirus mouse hepatitis virus JHM: sequence conservation within the surface glycoprotein gene S in Lewis rats. J Gen Virol.

[46] Nakatsukasa-Akune M, Yamashita K, Shimoda Y, Uchiumi T, Abe M, Aoki T, Kamizawa A, Ayabe S-i, Higashi S, Suzuki A (2005). Suppression of root nodule formation by artificial expression of the *TrEnodDR1* (coat protein of *White clover cryptic virus 2*) gene in. Lotus japonicus Mol Plant Microbe Interact.

[47] Ghabrial SA, Suzuki N, Granoff A, Webster R (2008). Fungal Viruses. Encyclopedia of Virology.

[48] Renault S, Stasiak K, Federici B, Bigot Y (2005). Commensal and mutualistic relationships of reoviruses with their parasitoid wasp hosts. J Insect Phys.

